# Neuroprotective effects of GLP-1 class drugs in Parkinson’s disease

**DOI:** 10.3389/fneur.2024.1462240

**Published:** 2024-12-10

**Authors:** Dongliang Lv, Peng Feng, Xueying Guan, Zhaona Liu, Dongfang Li, Cunshui Xue, Bo Bai, Christian Hölscher

**Affiliations:** ^1^Second Hospital, Shanxi Medical University, Taiyuan, China; ^2^Henan Academy of Innovations in Medical Science, Brain Institute, Zhengzhou, China

**Keywords:** Parkinson’s disease, diabetes, glucagon-like peptide 1, insulin, energy metabolism, neuroprotection

## Abstract

Parkinson’s disease (PD) is a chronic, progressive neurological disorder primarily affecting motor control, clinically characterized by resting tremor, bradykinesia, rigidity, and other symptoms that significantly diminish the quality of life. Currently, available treatments only alleviate symptoms without halting or delaying disease progression. There is a significant association between PD and type 2 diabetes mellitus (T2DM), possibly due to shared pathological mechanisms such as insulin resistance, chronic inflammation, and mitochondrial dysfunction. PD is caused by a deficiency of dopamine, a neurotransmitter in the brain that plays a critical role in the control of movement. Glucose metabolism and energy metabolism disorders also play an important role in the pathogenesis of PD. This review investigates the neuroprotective mechanisms of glucagon-like peptide-1 (GLP-1) and its receptor agonists, offering novel insights into potential therapeutic strategies for PD. GLP-1 class drugs, primarily used in diabetes management, show promise in addressing PD’s underlying pathophysiological mechanisms, including energy metabolism and neuroprotection. These drugs can cross the blood–brain barrier, improve insulin resistance, stabilize mitochondrial function, and enhance neuronal survival and function. Additionally, they exhibit significant anti-inflammatory and antioxidative stress effects, which are crucial in neurodegenerative diseases like PD. Research indicates that GLP-1 receptor agonists could improve both motor and cognitive symptoms in PD patients, marking a potential breakthrough in PD treatment and prevention. Further exploration of GLP-1’s molecular mechanisms in PD could provide new preventive and therapeutic approaches, especially for PD patients with concurrent T2DM. By targeting both metabolic and neurodegenerative pathways, GLP-1 receptor agonists represent a multifaceted approach to PD treatment, offering hope for better disease management and improved patient outcomes.

## 1 Introduction

Parkinson’s disease (PD) is one of the most disabling diseases globally (1). The etiology of PD is multifactorial. Currently, diagnosis is mainly based on its characteristic symptoms and signs, such as resting tremor, bradykinesia, and rigidity, which significantly reduce the patient’s quality of life. Although there are some medications and therapies available to alleviate the symptoms of PD, there is no cure for the disease. As PD progresses, patients eventually become severely disabled due to loss of motor control, imposing a heavy social burden (2–5). According to statistics, approximately 9.4 million people worldwide had PD in 2020, and the incidence is rising with global aging, especially in high-income and some middle-income countries (6, 7).

Type 2 diabetes mellitus (T2DM) is considered one of the risk factors for PD (8). Epidemiological studies have found that patients with T2DM have a significantly increased risk of developing PD (9–11). This association may be due to shared pathological mechanisms, including insulin resistance, chronic inflammation, and mitochondrial dysfunction (12). Additionally, abnormal insulin signaling pathways in T2DM patients may lead to neurodegeneration, increasing the risk of PD. Some experimental studies have also shown that diabetes medications, such as insulin sensitizers, may have protective effects on PD patients, further supporting the link between the two diseases. Currently, studies have found impaired insulin signaling and desensitization of insulin receptors in the brains of PD patients (13, 14). These phenomena have a complex relationship with the pathogenesis of Parkinson’s disease, as the onset of Parkinson’s disease is closely related to disturbances in glucose metabolism and energy conversion disorders. From these perspectives, research on diabetes medications may provide more possibilities for PD treatment.

## 2 Dopaminergic neurotransmission in PD pathogenesis

The dopaminergic neurotransmission in PD pathogenesis involves the loss and apoptosis of dopaminergic neurons in the brain, as well as a reduction in dopamine levels, leading to an absolute deficiency of dopamine (15). The exact cause of PD is not fully understood, but research suggests it may involve a combination of genetic and environmental factors. Most patients with Parkinson’s disease are over 60 years old, but there are cases where it appears at a younger age, known as early-onset PD. Dopamine (DA) is a crucial neurotransmitter. PD actually involves dysfunction in dopamine metabolism in many parts and regions of the brain, including synthesis, secretion, transport, reuptake, elimination, and receptor binding of DA (16). In dopaminergic neurons, tyrosine undergoes conversion to L-DOPA through the action of tyrosine hydroxylase (TH). Subsequently, L-DOPA is decarboxylated by aromatic L-amino acid decarboxylase (AADC) to produce DA. Most DA is transported to synaptic vesicle storage pools by vesicular monoamine transporter 2 (VMAT2) on the synaptic vesicle membrane, with a very low cytoplasmic DA concentration (17). When a nerve impulse reaches the synapse, it triggers the release of DA from synaptic vesicles into the synaptic cleft. Once released, DA binds to and activates dopamine receptors located on the postsynaptic membrane. The dopamine transporter (DAT) controls the extracellular concentration of DA, maintaining DA balance and regulating neurotransmitter signaling intensity. The more DAT, the stronger the ability to reuptake DA into the presynaptic membrane (18). DAT dysfunction can lead to dopamine system imbalance, causing related neurological diseases such as depression, attention deficit hyperactivity disorder, and cognitive impairment. Basic and clinical experiments have shown that reducing neurotransmitter reuptake by using transporter inhibitors, increasing the neurotransmitter content in the central synaptic cleft, is effective for treating neurological diseases (19). The degradation of free DA in dopaminergic neurons can proceed through two parallel pathways. In the first pathway, DA is deaminated by monoamine oxidase (MAO) to form 3,4-dihydroxyphenylacetaldehyde (DOPAL), which is then oxidized by aldehyde dehydrogenase (ALDH) to form 3,4-dihydroxyphenylacetic acid (DOPAC). Finally, DOPAC undergoes methylation by catechol-O-methyltransferase (COMT) to form homovanillic acid (HVA). In the second pathway, DA is first converted to 3-methoxytyramine (3-MT) by COMT, and then sequentially acted upon by MAO and ALDH to eventually produce HVA (20, 21). Disruption of DA homeostasis leads to a reduction in DA, exacerbating PD symptoms, which is the basic mechanism of PD pathogenesis.

## 3 PD pathogenesis and glucose metabolism and energy conversion disorders

The pathogenesis of PD is linked to disturbances in glucose metabolism and energy conversion disorders. PD is closely related to energy metabolism imbalances in the dopamine system. PD patients generally have lower energy metabolism levels and lower efficiency of energy conversion in the body. A study found that the basal metabolic rate of PD patients was significantly reduced, indicating a lower level of energy consumption. Additionally, motor dysfunction in PD patients leads to reduced physical activity, further affecting energy consumption and metabolism (22). These findings suggest the presence of energy conversion disorders in PD patients, which may be closely related to the pathophysiological mechanisms of the disease. Current research has shown that mild cognitive impairment in Parkinson’s patients is associated with brain energy metabolism (23). The function of the dopamine system is highly complex, and the processes of dopamine synthesis, secretion, transport, reuptake, elimination, and receptor binding are all closely related to energy metabolism. Mitochondrial dysfunction, insulin resistance, and glucose metabolism abnormalities collectively lead to insufficient energy supply and neuronal damage, with insulin resistance and mitochondrial dysfunction being key pathways affecting energy metabolism imbalance in PD patients.

### 3.1 PD and insulin resistance

Insulin resistance (IR) is usually considered a systemic response or reduced sensitivity to insulin effects. IR is not only a marker of T2DM but also a significant factor leading to PD. Compared to age-matched controls, insulin resistance in the brains of PD patients is independent of peripheral insulin resistance and manifests as severe abnormalities in brain insulin signal transduction. Patients with PD primarily exhibit a significant loss of insulin receptor mRNA in the substantia nigra pars compacta, increased insulin IR, and reduced activation of molecules such as insulin receptor substrate 1 (IRS1) and protein kinase B (Akt) within the insulin metabolic pathway (24). On one hand, IR leads to abnormal glucose uptake and oxidation, reduced glycogen synthesis, and decreased ability to inhibit lipid metabolism, resulting in glucose metabolism imbalance. Glucose homeostasis abnormalities can lead to chronic hyperglycemia, causing oxidative stress. Oxidative stress activates microglia and reactive astrocytes, leading to inflammation and subsequent cellular damage (25). In summary, brain IR in PD ultimately leads to dopaminergic cell damage by affecting energy metabolism. On the other hand, the major pathological changes in PD include abnormal protein aggregation, neuroglial dysfunction, oxidative stress, glutamate toxicity, calcium overload, and mitochondrial dysfunction. Abnormalities in energy metabolism may underlie the vulnerability of dopaminergic neurons in the substantia nigra of PD patients (26). Mitochondrial dysfunction, improper protein processing, and oxidative stress are direct consequences of metabolic abnormalities; oxidative stress, calcium, and glutamate toxicity overload may also lead to energy deficiencies, forming a vicious cycle with *α*-synuclein accumulation.

Under normal circumstances, insulin maintains the NAD(+)/NADH ratio by activating the phosphoinositide 3-kinase/protein kinase B (PI3K/AKT) pathway and inhibiting the forkhead box protein O1/heme oxygenase 1 (Foxo1/Hmox1) pathway, thereby mediating the activation of the sirtuin 1/peroxisome proliferator-activated receptor-gamma coactivator 1-alpha (SIRT1/PGC-1α) pathway and regulating mitochondrial physiological functions (27). Additionally, another downstream substrate of the insulin-induced PI3K/AKT pathway is serine phosphorylation at the 133rd position of CREB, which increases mitochondrial membrane potential, hexokinase activity, NAD(P)H redox state, and intracellular ATP levels (28). When insulin resistance occurs, mitochondrial-related protein levels change, mitochondrial membrane potential decreases, calcium homeostasis is disrupted (29), and reactive oxygen species (ROS) production increases, leading to cell death. Normal insulin function is closely related to mitochondrial function and dynamic balance, and insulin resistance directly leads to mitochondrial dysfunction. Therefore, IR is a new effective target for systemic PD treatment, especially in the brain (30).

### 3.2 PD and mitochondrial dysfunction

Mitochondria are the most common organelles in eukaryotic cells, generating energy through cellular oxidative phosphorylation, converting adenosine diphosphate (ADP) to adenosine triphosphate (ATP), and providing energy for neurons. Mitochondria play a crucial role in electron transport and oxidative phosphorylation (31). Increasing evidence suggests that mitochondrial dysfunction is central to many neurodegenerative diseases, such as PD, Alzheimer’s disease (AD), amyotrophic lateral sclerosis (ALS), and Huntington’s disease (HD) (32–37).

During oxidative phosphorylation, the ROS produced by normally functioning cells must be kept at low levels. Mitochondrial dysfunction is a central feature of PD pathogenesis, especially through interference with Complex I and Complex II of the electron transport chain (ETC). This dysfunction leads to reduced ATP production and increased ROS formation, which results in oxidative stress and neuronal damage. Studies have shown that Complex I activity is diminished in both post-mortem brain tissue and peripheral blood cells of PD patients (38, 39). A new preclinical study at Northwestern University’s Feinberg School of Medicine found that disrupting complex I function in mouse dopaminergic neurons led to progressive PD-related motor deficits, with loss of dopamine release in the substantia nigra being critical for motor deficits (40).

Previous studies have shown that mitochondrial dysfunction related to PD can be caused by various factors, including mitochondrial bio-genetic defects, increased ROS production, mitophagy defects, transport disorders, electron transport chain dysfunction, mitochondrial dynamics changes, and mitochondrial calcium imbalance. If this dysfunction is not promptly blocked, it will eventually lead to cell damage and death (41). Substantia nigra-striatal neurons have higher energy demands than other cells, making them more susceptible to mitochondrial dysfunction (42). Some studies have shown reduced expression of major components of the electron transport chain in the striatum, frontal cortex, and substantia nigra of PD patients (43). The mitochondrial oxidative phosphorylation system comprises the ETC and ATP synthase, with mitochondrial respiratory chain complex I (CI) being a crucial component located on the inner mitochondrial membrane. Complex I dysfunction is considered a major factor in PD pathogenesis (44). ROS in cells mainly originate from mitochondrial complexes I and III (45). ETC abnormalities lead to significant loss of mitochondrial bioenergetic function and oxidative stress, increasing neurons’ susceptibility to excitotoxic damage, leading to PD (46). MPP+, a metabolite of MPTP, is a complex I inhibitor. When MPP+ enters cells, it inhibits mitochondrial complex I enzyme and NADH–ubiquinone oxidoreductase, causing ROS formation and mitochondrial electron leakage (47). MPP + -induced oxidative stress promotes *α*-synuclein accumulation and misfolding in the brain, leading to neurotoxicity (48). These findings illustrate the role of mitochondrial defects in increasing oxidative stress and reducing ATP synthesis, which leads to increased neuronal vulnerability. Toxins like MPP+ and rotenone, which inhibit Complex I, cause a buildup of ROS, reinforcing the connection between ETC dysfunction and disease progression in PD.

Changes in mitochondrial dynamics include the highly dynamic process of fission-fusion. Mitochondrial fission is controlled by dynamin-related protein 1 (Drp1) and mitochondrial fission protein 1 (FIS1). When mitochondria signal for mitosis, Drp1 translocates from the cytoplasm to the outer mitochondrial membrane, interacts with FIS1, and forms a spiral structure around the mitochondria to split them (49). Compared to fission, mitochondrial fusion is regulated by mitochondrial fusion proteins 1/2 (Mfn1/2) and optic atrophy 1 (OPA1), which control the fusion of the inner mitochondrial membrane (IMM) and outer mitochondrial membrane (OMM), respectively (50). Mitochondrial fission is associated with apoptosis (51), and increased fission can lead to mitochondrial fragmentation, reduced membrane potential, and decreased ATP production. Mitochondrial fusion complements the exchange of proteins, respiratory chain complexes, and mitochondrial DNA (mtDNA). Impaired fusion leads to increased mutation rates and genome loss, resulting in deficiencies in oxidative phosphorylation (OXPHOS) and significantly elevated reactive ROS (52), causing insufficient energy uptake and death of dopaminergic neurons. These findings highlight the role of mitochondrial dysfunction in PD and suggest that targeting mitochondrial pathways may provide neuroprotection.

**Figure 1:**
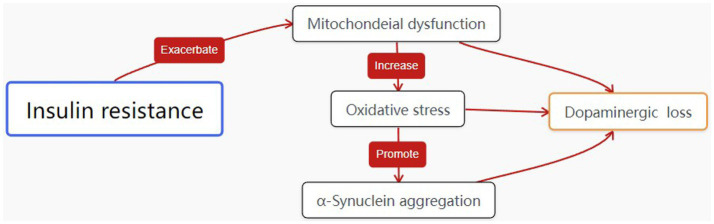
The interplay between energy metabolism abnormalities, dopamine neurotrasmettitorial impairment, PD pathogenesis and insulin resistance.

The relationship between energy metabolism abnormalities, dopamine neurotransmission impairment, and insulin resistance is complex and multifaceted. PD patient’s exhibit reduced energy metabolism due to mitochondrial dysfunction, which leads to decreased dopamine synthesis and increased oxidative stress. Insulin resistance further exacerbates this dysfunction by impairing glucose metabolism, resulting in an energy deficit for neurons (53, 54). Studies have shown that insulin resistance in the brain contributes to dopaminergic neuron vulnerability, promoting oxidative stress, mitochondrial dysfunction, and protein aggregation (55). In this respect the complex relationship is similar to that shown in Figure 1.On the other hand, energy conversion disorders may exacerbate glucose metabolism disorders. Inefficient energy metabolism affects insulin sensitivity and insulin-like growth factor function, impacting blood glucose regulation. Therefore, in-depth research into the relationship between energy metabolism abnormalities, dopamine neurotrasmettitorial impairment, and insulin resistance will help us better understand the PD pathogenesis. Additionally, apart from IR being a new effective target for systemic PD treatment, studies on T2DM have found that ROS production and mitochondrial damage in muscle tissues of hyperglycemic mice can be normalized with antioxidant treatment and blood glucose control (56), suggesting that T2DM treatment strategies could provide new insights and strategies for future PD research and treatment.

## 4 Glucagon-like peptide-1

Incretins are polypeptide hormones secreted by enteroendocrine cells that promote insulin secretion. GLP-1 and glucose-dependent insulinotropic polypeptide (GIP) are the primary members (57). Enteroendocrine L-cells secrete GLP-1 in response to various nutrients after food intake, releasing it into the bloodstream. GLP-1 binds to its specific receptor (GLP1R), promoting insulin release. Additionally, GLP-1 activates related intracellular signaling pathways, promoting *β*-cell regeneration, inhibiting β-cell apoptosis, and improving β-cell function, thereby lowering blood glucose levels, with its effects being glucose concentration-dependent (58, 59). When blood glucose levels return to normal, the incretin effect diminishes, maintaining blood glucose at normal levels with high safety (60, 61). T2DM patients often suffer from comorbid conditions such as cardiovascular disease, and GLP-1 not only has a good glucose-lowering effect but also has protective effects on the cardiovascular system, kidneys, and other tissues (62, 63). GIP, a sister incretin hormone to GLP-1, also plays a role in glucose homeostasis.

In recent years, research has discovered the potential effects of GLP-1 and its analogs on the nervous system, particularly in relation to the blood–brain barrier (BBB). Firstly, GLP-1 and its analogs can cross the BBB (64, 65). GLP-1 receptors are widely distributed in the central nervous system, especially in regions related to appetite regulation, memory, and learning, including the hippocampus, hypothalamus, and brainstem. By activating these receptors, GLP-1 exerts various biological effects in the central nervous system, including promoting neuroprotection and neurogenesis. Secondly, GLP-1 and its analogs protect BBB integrity. The BBB is a critical structure maintaining central nervous system homeostasis, blocks harmful substances from entering the brain while permitting essential nutrients to pass through. Studies have shown that GLP-1 reduces BBB permeability, decreases inflammation, and enhances structural stability, protecting the brain from external harmful substances. Moreover, the anti-inflammatory and antioxidant effects of GLP-1 also help protect the BBB (66–68). In neurodegenerative diseases such as PD and AD, BBB dysfunction is a common pathological feature. Clinical trials have tested the effects of GLP-1R agonists in patients with PD, AD, or diabetes-related memory impairment, showing significant improvement in these patients’ conditions (69). GLP-1 may help improve BBB function in these diseases by reducing oxidative stress and inflammation.

The neuroprotective effects of GLP-1R agonists may be closely related to their impact on insulin resistance and glucose metabolism. Insulin resistance and glucose metabolism disorders are not only associated with metabolic diseases like diabetes but also closely linked to the onset and progression of neurodegenerative diseases. GLP-1R agonists, by regulating insulin release and lowering blood glucose levels, may help improve insulin resistance and glucose metabolism disorders, indirectly protecting the nervous system from damage.

In summary, GLP-1 not only plays an important role in peripheral glucose regulation but also crosses the BBB, protecting and regulating BBB function through various mechanisms. Further research may provide new insights and methods for the application of GLP-1 and its analogs in treating neurological diseases.

## 5 Research on the neuroprotective effects of GLP-1

GLP-1 has neuroprotective properties and growth factor-like, promoting mitosis, cell growth, and differentiation, blocking pre-apoptotic processes, protecting neurons from oxidative stress and endoplasmic reticulum stress, and promoting endothelial cell proliferation and angiogenesis. Currently, its neuroprotective effects on neurodegenerative diseases such as AD and PD have been widely studied. In some animal and clinical trials, GLP-1 analogs (Exendin-4, Liraglutide) have been shown to have neuroprotective effects on PD and AD (70–72). Additionally, another major member of incretins, GIP, and its analogs have also demonstrated neuroprotective effects in disease animal models and can enhance the effects of GLP-1. GIP analogs (D-Ala2-GIP) can directly regulate neurotransmitter release and promote hippocampal stem cell proliferation, showing varying degrees of neuroprotective effects in AD and PD mouse models (73, 74).

**Figure 2:**
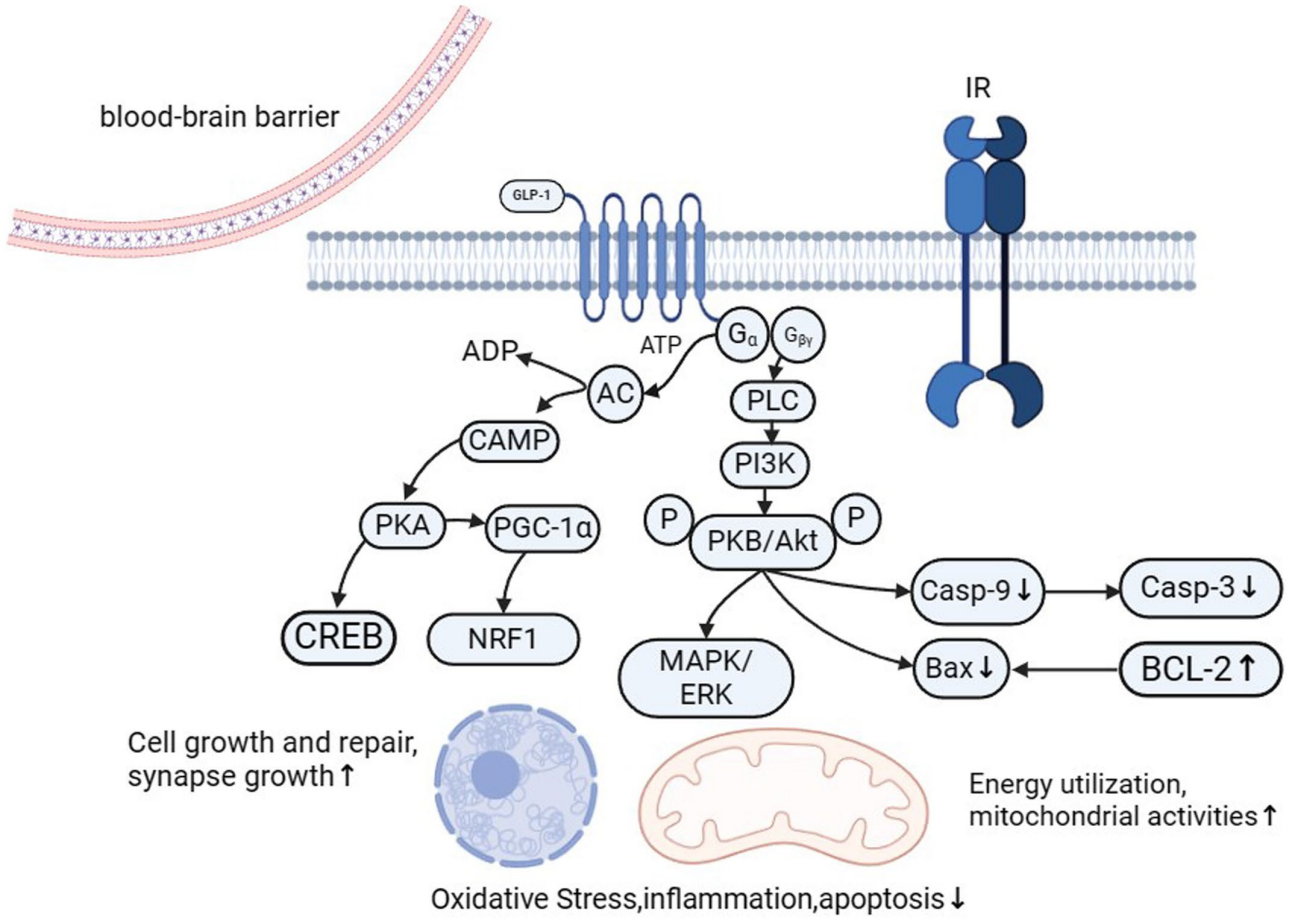
GLP-1 (glucagon-like peptide-1) plays a vital role in neuroprotection and regeneration through multiple mechanisms. It promotes the expression of NGF and BDNF, activates the PI3K/Akt signaling pathway, and reduces neuroinflammation, thereby facilitating nerve regeneration. GLP-1 also enhances mitochondrial activity by activating AMPK, increasing the expression of genes involved in mitochondrial biogenesis, and reducing the activation of mitochondrial apoptotic pathways. It reduces oxidative stress by decreasing ROS production and upregulating antioxidant enzymes. Additionally, GLP-1 inhibits the NF-κB signaling pathway and reduces the activation of microglia and astrocytes, thereby mitigating inflammatory responses. Furthermore, GLP-1 significantly decreases cell apoptosis by regulating the expression of Bcl-2 family proteins and inhibiting apoptotic signaling pathways. These combined mechanisms enable GLP-1 to play a vital protective role in maintaining the health and function of the nervous system.

.Given the correlation between PD and glucose metabolism disorders and energy conversion disorders, researchers have increasingly focused on the neuroprotective effects of GLP-1.GLP-1 receptor agonists provide neuroprotection through multiple pathways, including improving insulin sensitivity, reducing oxidative stress, and enhancing mitochondrial function, as shown in Figure 2. These drugs activate key signaling pathways, such as PI3K/Akt and AMPK, which enhance mitochondrial biogenesis and promote neuronal survival. In patients with PD and coexisting type 2 diabetes, GLP-1 receptor agonists offer dual therapeutic benefits, addressing both metabolic and neurodegenerative mechanisms. This dual-action makes GLP-1 receptor agonists (GLP-1 Ras) a promising treatment strategy for PD.

The neuroprotective effects of GLP-1 receptor agonists (RAs) such as liraglutide and exendin-4 have been demonstrated in numerous clinical trials related to diabetic neurological complications, including stroke and cognitive impairment (75–78). Several clinical trials have evaluated the efficacy of GLP-1 RAs in PD. A open-label trial tested exenatide as a potential treatment for PD in a non-randomized, open-label study with 45 patients. Patients were administered exenatide for 48 weeks, followed by a 12-week washout period. The trial showed that patients treated with exenatide exhibited significant improvements in motor function (measured by the UPDRS motor score) that persisted even after stopping the drug. The study also indicated potential neuroprotective effects, with no significant progression of PD during the trial (79). And then leveraging the neuroprotective properties of GLP-1 receptor agonists, researchers initiated a Phase II clinical trial to evaluate exenatide therapy for PD. In this trial, at 60 weeks, the exenatide group showed a significant improvement in MDS-UPDRS Part III scores compared to the placebo group among the 62 recruited patients (80). In another Phase II clinical trial also using exenatide, 60 patients participated, with 29 in the placebo group and 31 in the exenatide group. Compared to the placebo group, patients receiving exenatide treatment exhibited increased tyrosine phosphorylation of insulin receptor substrate 1 at both 48 and 60 weeks. This study suggests that using neuron-derived extracellular vesicles (EVs) extracted and enriched from peripheral blood samples as biomarkers has potential for assessing molecular responses to therapeutic interventions in clinical trials for neurological diseases. The results indicate that exenatide treatment may be associated with enhanced brain insulin signaling pathways. More importantly, PD patients demonstrated motor improvements even after stopping the medication (81, 82). The Cochrane systematic review by Mulvaney et al. (83) reported significant improvements in motor function, as well as potential reductions in non-motor symptoms in PD patients treated with exenatide (83). Additionally, a study by Kalinderi et al. (84) highlighted the neuroprotective potential of GLP-1 RAs, particularly in reducing protein aggregation and improving mitochondrial function. These findings suggest that GLP-1 receptor agonists may slow disease progression through multiple mechanisms, including improved insulin sensitivity and energy metabolism (84). A recent phase 2 clinical trial testing the GLP-1 class drug lixisenatide showed clear improvements in PD patients in MDS-UPDRS Part III scores and stopped disease progression (85). There are still phase 3 trials of drugs such as exenatide in PD that are either ongoing or are analyzing data (86).

In other respects, a retrospective cohort study aimed to assess the neurological and psychiatric outcomes of semaglutide compared to other antidiabetic drugs (sitagliptin, empagliflozin, and glipizide) over 12 months in patients with type 2 diabetes (T2DM). The study used electronic health records (EHRs) from the TriNetX US Collaborative Network, covering over 100 million patients. Propensity-score matching was applied to control for baseline differences between cohorts, ensuring comparability. The analysis focused on 22 neurological and psychiatric outcomes, including cognitive deficits, dementia, parkinsonism, psychosis, and substance misuse. Cox regression analysis was used to compare the risks between semaglutide and the comparator drugs. The study found that semaglutide use was not associated with an increased risk of neurological or psychiatric conditions compared to the other antidiabetic drugs. Notably, semaglutide was associated with a reduced risk of cognitive deficits and dementia compared to sitagliptin and glipizide, though no significant difference was found when compared to empagliflozin. Semaglutide also showed a lower risk of nicotine misuse across comparisons, though results varied with statistical correction. These findings suggest potential neuroprotective benefits of semaglutide, especially regarding cognitive health, and call for further research to validate its use in treating cognitive deficits and substance misuse in patients with T2DM (87). In summary, an increasing number of clinical studies support that GLP-1 RAs can improve the clinical and pathological changes in PD, providing long-term benefits for patients’ cognitive and motor symptoms. Therefore, we speculate that applying incretin-based medications in PD models can promote insulin secretion, improve peripheral and central insulin resistance, stabilize mitochondrial function, enhance energy metabolism, and exert neuroprotective effects that stabilize dopamine homeostasis (88). The specific mechanisms and long-term effects of GLP-1 class drugs in PD are worthy of further clinical trials.

### 5.1 GLP-1’s role in improving energy metabolism

GLP-1 RAs play a vital role in enhancing energy metabolism by boosting mitochondrial function and regulating glucose and lipid metabolism. Activation of the PI3K/Akt and AMPK signaling pathways leads to increased mitochondrial biogenesis and enhanced energy production, which is particularly important in neurodegenerative conditions like PD (89–91). GLP-1 RAs also improve glucose uptake and utilization, ensuring energy stability even in metabolically challenged neurons (92). This protective effect reduces mitochondrial dysfunction, a key driver of neurodegeneration (93).

### 5.2 GLP-1’s antioxidative stress effects

GLP-1 RAs have robust antioxidative properties, primarily through the activation of the Nrf2 pathway, which upregulates antioxidant enzymes like superoxide dismutase (SOD) and catalase. These enzymes reduce the levels of reactive oxygen species (ROS), mitigating oxidative stress in neurons (94–96). By activating AMPK, GLP-1 RAs improve mitochondrial function, further reducing ROS production and preventing oxidative stress-induced apoptosis (97). This antioxidative action is crucial for protecting neurons in PD, where oxidative damage is a significant contributor to disease progression (98).

### 5.3 GLP-1’s anti-inflammatory effects

GLP-1 receptor agonists significantly reduce inflammation by modulating immune cell activity and suppressing the release of pro-inflammatory cytokines, such as TNF-*α*, IL-6, and IL-1β (99–101). These cytokines play a major role in neuroinflammation, which exacerbates neuronal damage in neurodegenerative diseases like PD. Additionally, GLP-1 RAs inhibit the NF-κB pathway, which is central to the regulation of inflammatory responses (102, 103). By dampening inflammation and modulating microglial activity, GLP-1 RAs create a neuroprotective environment that supports neuronal survival.

### 5.4 GLP-1’s anti-apoptotic effects

GLP-1 RAs exhibit strong anti-apoptotic effects by activating the PI3K/Akt pathway, which promotes cell survival by inhibiting pro-apoptotic factors such as Bax and caspase-3, while upregulating anti-apoptotic proteins like Bcl-2 (104–106). In addition, GLP-1 RAs protect against mitochondrial dysfunction, preventing the release of cytochrome c and stabilizing mitochondrial membranes, thereby inhibiting apoptosis. This mitochondrial protection is especially critical in PD, where neuronal loss is driven by apoptotic processes.

### 5.5 GLP-1’s role in promoting neurogenesis

GLP-1 RAs have been shown to promote neurogenesis by stimulating the proliferation of neural progenitor cells (NPCs) and supporting their differentiation into functional neurons. This process is mediated through the PI3K/Akt pathway, which enhances neurogenesis and promotes neuronal survival. GLP-1 RAs also modulate the brain’s microenvironment by reducing neuroinflammation, allowing for more efficient neurogenesis. Additionally, these agents increase the expression of neurotrophic factors such as brain-derived neurotrophic factor (BDNF) and nerve growth factor (NGF), which support the survival and maturation of new neurons (107).

### 5.6 Protein aggregation

*α*-Synuclein aggregation is a hallmark of PD, forming Lewy bodies that contribute to neuronal death, particularly in dopaminergic neurons in the substantia nigra. Misfolded α-synuclein proteins accumulate, leading to mitochondrial dysfunction, oxidative stress, and inflammation, all of which drive neurodegeneration (108). Given that α-synuclein aggregation plays such a central role in PD pathology, any intervention that can reduce or prevent this process offers a potential disease-modifying treatment. GLP-1 receptor agonists are showing promise in this area, which elevates their potential beyond symptomatic relief to a more foundational disease-modifying approach.

Preclinical models, particularly those using MPTP-induced PD models in rodents, have demonstrated that GLP-1 RAs can reduce the accumulation of α-synuclein. For example, in studies with exendin-4, researchers observed reduced α-synuclein aggregation and improved neuronal survival in the substantia nigra. These findings are encouraging as they indicate that GLP-1 RAs can intervene in one of the root causes of PD neurodegeneration. In clinical studies, while the direct effects on α-synuclein aggregation in humans are still being investigated, improvements in motor function, cognitive abilities, and general neuronal health have been observed, suggesting that GLP-1 RAs are impacting these pathological processes.

## 6 Research on other GLP-1 related drugs

Currently, research on the neuroprotective effects of GLP-1/GIP dual receptor agonists in PD is continuously advancing. Studies have shown that the first-generation dual receptor agonist (DA-JC1) has neuroprotective effects on MPTP-induced PD mice. Results indicate that DA-JC1 reduces neuroinflammation by inhibiting the proliferation of astrocytes and microglia in the substantia nigra, and mitigates the damage of toxic substances MPTP to midbrain dopaminergic neurons and fibers by promoting the expression of BDNF, phosphorylation of Akt in the PI3K/Akt signaling pathway, downregulating the pro-apoptotic protein Bax, and upregulating the anti-apoptotic protein Bcl2 (109, 110).

**Figure 3:**
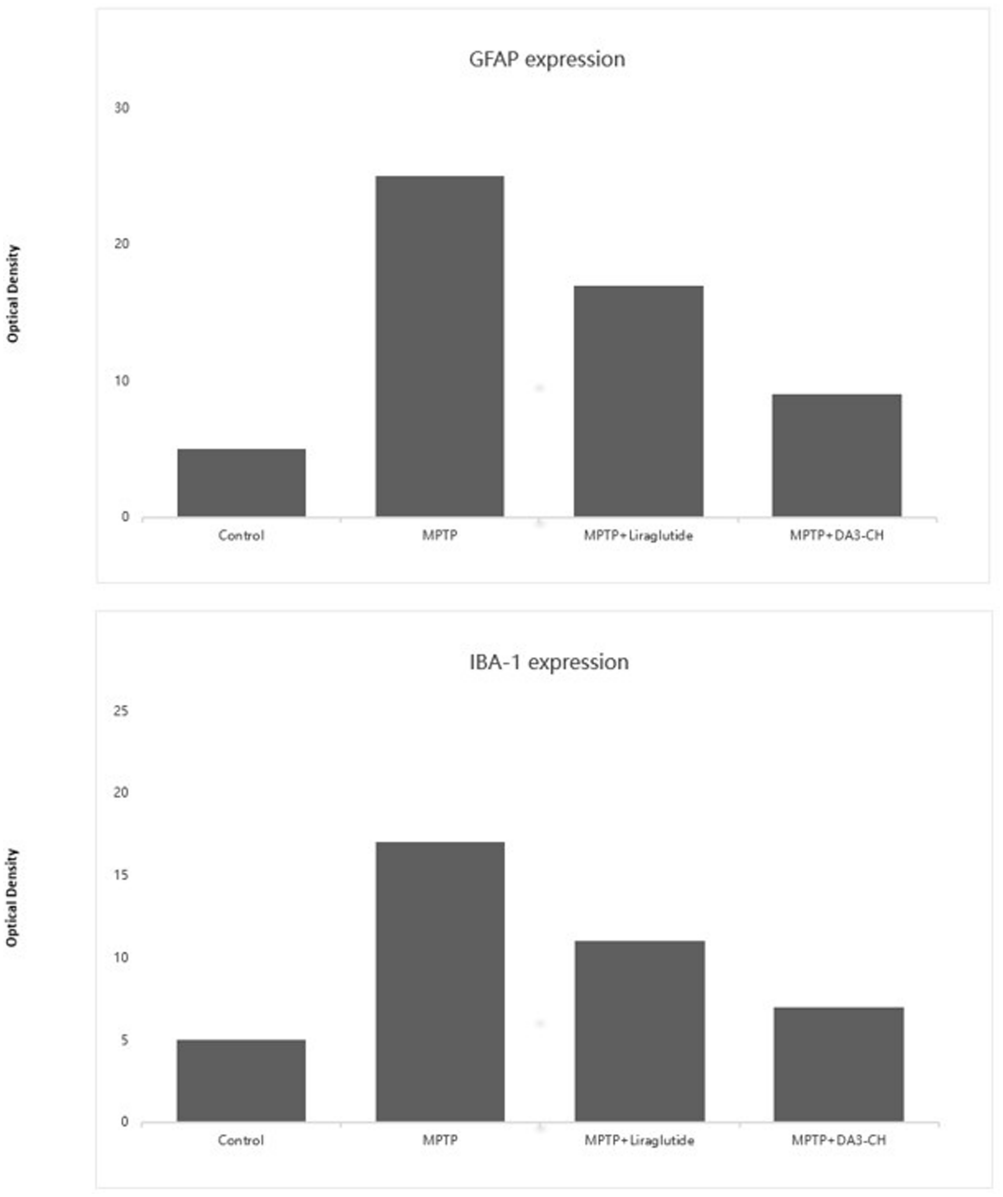
GFAP serves as a marker of astrocyte activation. The data indicate that after MPTP treatment, there is a significant increase in GFAP expression, signifying pronounced astrocytic activation and an inflammatory response. However, in the DA3 and liraglutide treatment groups, the expression levels of GFAP are significantly decreased compared to the MPTP group (*p* < 0.01), suggesting that these treatments may exert anti-inflammatory effects by reducing astrocyte activation. IBA-1 is a marker of microglial activation. Compared to the control group, MPTP treatment significantly increases the expression of IBA-1 (*p* < 0.01), indicating the activation of microglia. In the DA3 and liraglutide treatment groups, the expression levels of IBA-1 are significantly reduced compared to the MPTP group (*p* < 0.001), demonstrating that these treatments effectively inhibit excessive microglial activation. Additionally, the reduction in IBA-1 expression is more pronounced in the DA3 + MPTP group compared to the liraglutide+MPTP group (*p* < 0.01). These findings highlight the potential of DA3 and liraglutide to modulate the expression of inflammatory markers, possibly playing a crucial role in their mechanisms of action against neuroinflammation. The reduction in both GFAP and IBA-1 levels suggests effective anti-inflammatory properties, further supporting their use in therapeutic strategies aimed at mitigating the pathological features of neurodegenerative diseases such as PD. Figure modified from Yuan et al. (111).

.An experiment applied a second-generation novel GLP-1/GIP dual receptor agonist, DA3-CH, to an MPTP-induced Parkinson’s mouse model and compared its neuroprotective effects with the GLP-1 single receptor agonist, liraglutide. DA-JC1 was confirmed to have neuroprotective effects in the MPTP-induced PD mouse model; however, it did not show superior effects compared to Liraglutide. Based on this, the experiment used another new dual receptor agonist. The results showed that both DA3-CH and Liraglutide could reduce MPTP-induced neuroinflammatory responses, inhibit the proliferation of microglia and astrocytes, and DA3-CH had significantly better effects than Liraglutide, as shown in Figure 3 (111).

**Figure 4:**
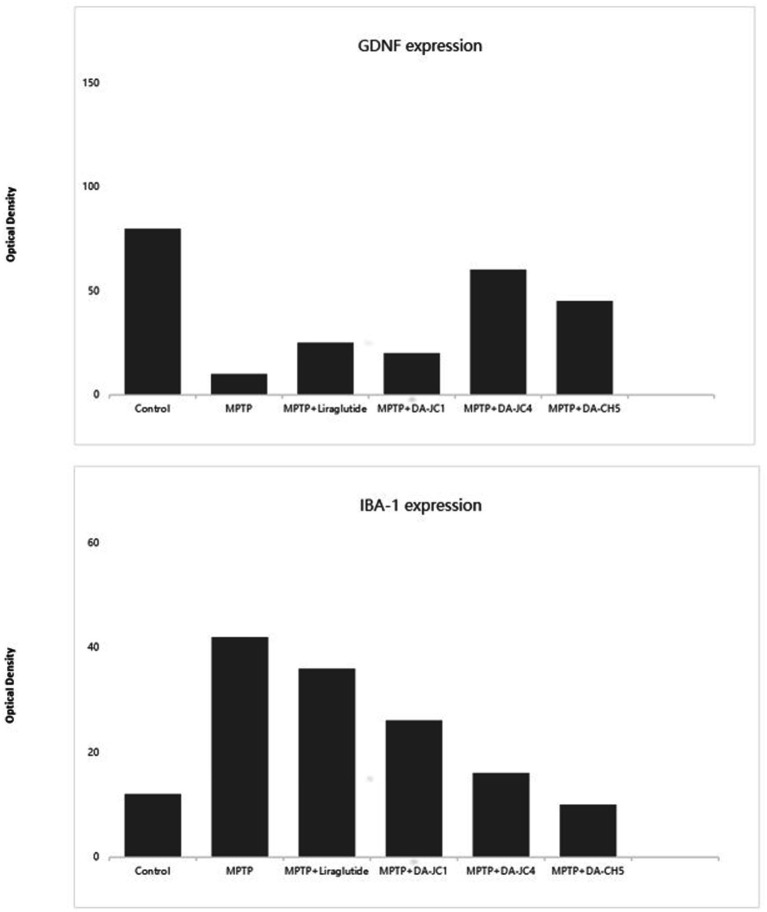
The figure presents a quantitative comparison of the number of GDNF-positive cells in the substantia nigra pars compacta across different treatment groups and the quantity of IBA-1-positive glial cells in the substantia nigra pars compacta across different treatment groups. A one-way ANOVA revealed significant differences between groups (*F* = 78.736; *p* = 0.03). Subsequent Tukey post-hoc tests highlighted significant differences: Compared to the control group, all treatment groups (MPTP, DA-CH5, and other dual GLP-1/GIP agonist treatment groups) showed a significant increase in the number of GDNF-positive cells, demonstrating these treatments’ effectiveness in maintaining or promoting the expression of GDNF. Compared to the MPTP-treated group, the DA-CH5 treated group showed the most significant increase in GDNF-positive cells, indicating that DA-CH5 was the most effective treatment among those tested. Additionally, the effects of DA-CH5 were more pronounced compared to the liraglutide group, showcasing its unique neuroprotective potential. The one-way ANOVA indicated that while the overall differences between groups did not reach traditional levels of statistical significance (*p* = 0.156), trends suggest potential biologically meaningful differences between certain groups. Compared to the control group, MPTP treatment significantly increased the number of IBA-1-positive glial cells, indicating an increase in inflammation. In the DA-CH5 treated group, the number of IBA-1-positive glial cells was significantly reduced compared to the MPTP group, suggesting anti-inflammatory effects of DA-CH5. Furthermore, DA-CH5 showed superior performance in reducing IBA-1-positive glial cells compared to the liraglutide group, highlighting its potential anti-inflammatory and neuroprotective effects. These results emphasize the potential advantages of DA-CH5 and other dual agonists in enhancing the expression of the neurotrophic factor GDNF, potentially offering new strategies for treating PD and suggest that DA-CH5 not only enhances the expression of neurotrophic factors but also effectively reduces inflammation associated with PD, providing strong evidence for its potential in clinical applications. Figure modified from Feng et al. (112).

In another study comparing the neuroprotective effects of two other novel GLP-1/GIP dual receptor agonists (DA-JC4, DA-CH5) with the GLP-1 single receptor agonist liraglutide and the first-generation GLP-1/GIP dual receptor agonist DA-JC1 in MPTP-induced PD mice, the results concluded that the new dual receptor agonists have clear neuroprotective effects (112). They showed better effects in improving mouse motor function scores, promoting the repair and regeneration of synapses or dopaminergic neurons (TH+, GDNF, synaptophysin), and inhibiting neuroinflammatory responses (IBA-1, IL-2) compared to liraglutide and DA-JC1, highlighting the advantage of targeting more sites. Figure 4 illustrates some of the findings. Comparative studies between the two GLP-1/GIP dual receptor agonists showed that DA-CH5 had better effects in promoting TH+ expression and reducing IL-2 and TNF expression than DA-JC4, with no significant differences in other comparisons. This indicates that DA-CH5 has a greater comprehensive advantage and more optimal target sites, making it more likely to be used clinically in the future compared to DA-JC4 and DA-JC1.

Due to the influence of renal clearance and dipeptidyl peptidase-4 (DPP-4), GLP-1 has a short natural half-life in the human body, about 1–2 min (113). Therefore, developing DPP-4 inhibitors or GLP-1 analogs has become a primary means of enhancing GLP-1 efficiency in the body. Most incretin-based drugs are based on GLP-1. Approved drugs for diabetes treatment, including GLP-1 or GIP analogs and their receptor agonists, have been widely used in neurodegenerative disease research, such as Exenatide and Liraglutide. These analogs are eight times more potent than endogenous GLP-1 (114). Research on multi-receptor agonists of incretins may further develop the neuroprotective effects of GLP-1 and related drugs.

## 7 Discussion

Current research suggests a close relationship between glucose metabolism disorders and energy conversion disorders and PD pathogenesis. On one hand, IR leads to abnormal glucose uptake and oxidation, reduced glycogen synthesis, decreased lipid metabolism inhibition, resulting in glucose metabolism imbalance. Glucose homeostasis abnormalities can cause chronic hyperglycemia, triggering oxidative stress. Oxidative stress activates microglia and reactive astrocytes, inducing inflammation, leading to cellular damage. This chronic inflammatory state further exacerbates neuronal damage and death, creating a vicious cycle. On the other hand, major pathological changes in PD include abnormal mitochondrial dysfunction, protein aggregation, oxidative stress, glutamate toxicity, calcium overload, and neuroglial dysfunction. Mitochondrial dysfunction, improper protein processing, and oxidative stress are direct consequences of metabolic abnormalities; oxidative stress, calcium overload, and glutamate toxicity may also lead to energy deficiencies, forming a vicious cycle with *α*-synuclein accumulation. Autopsy results of PD patients reveal a significant loss of dopaminergic neurons in the substantia nigra pars compacta, while the olfactory bulb and ventral tegmental area are less affected. This may be due to the higher oxidative stress levels and basal metabolic rate of dopaminergic neurons in the substantia nigra pars compacta. In conclusion, research findings suggest that energy metabolism abnormalities may be the cause of the vulnerability of dopaminergic neurons in the substantia nigra of PD patients. Therefore, studying the impact of glucose metabolism disorders and energy conversion disorders on dopamine homeostasis may bring new progress in exploring the etiology of PD.

Both T2DM and PD share insulin resistance as a contributing factor in disease progression. In T2DM, insulin resistance leads to poor glucose control, while in PD, insulin resistance within the brain can exacerbate neurodegeneration by impairing glucose uptake and metabolism. GLP-1 RAs improve insulin sensitivity and glucose metabolism, offering benefits for both peripheral metabolic control (T2DM) and central glucose metabolism in the brain (PD). By targeting insulin resistance, GLP-1 RAs may reduce the toxic effects of hyperglycemia on neurons and prevent the chronic inflammation associated with both diseases. This dual action of improving systemic insulin resistance and acting on brain insulin receptors makes GLP-1 RAs a unique treatment option for individuals with both conditions. At the same time, GLP-1 RAs have shown promise in improving cognitive function, not only in diabetes but also in neurodegenerative diseases like PD and Alzheimer’s disease. This cognitive benefit is especially relevant for T2DM patients with PD, as cognitive decline is a common symptom in both conditions.

Meanwhile, new GLP-1/GIP dual receptor agonists have been proven effective in neuroprotection and mechanisms in PD, such as DA-JC1 (DA1), DA-JC4 (DA4), and DA-CH5 (DA5) in MPTP-induced PD models. They significantly inhibit apoptosis, anti-inflammatory responses, protect synaptic function, promote the release of neurotrophic factors, and outperform GLP-1 single receptor agonists. Additionally, GLP-1 receptor agonists not only play an essential role in glucose metabolism and energy conversion but also exhibit significant anti-inflammatory and antioxidative stress effects. Research shows that GLP-1 effectively reduces neuroinflammation by decreasing pro-inflammatory cytokine release and inhibiting microglia activation. This anti-inflammatory effect is crucial for protecting neurons and slowing the progression of neurodegenerative changes. Simultaneously, GLP-1 receptor agonists enhance cellular antioxidant capacity by activating antioxidant enzyme systems, reducing ROS production, further protecting neurons from oxidative stress damage.

More importantly, GLP-1 receptor agonists also show potential in promoting neurogenesis. Studies have found that GLP-1 promotes neural stem cell proliferation and differentiation, increasing dopaminergic neuron generation by activating PI3K/Akt and MAPK/ERK signaling pathways. This may provide new therapeutic strategies for restoring the damaged dopaminergic system in PD patients.

In conclusion, given the unclear etiology of PD in clinical practice, further exploration of the molecular mechanisms by which GLP-1 and its analogs influence mitochondrial function and dopamine homeostasis in PD treatment may provide new insights for preventing PD in T2DM patients and offer potential targets for clinical relief and treatment of PD. Research on GLP-1 receptor agonists and new GLP-1/GIP dual receptor agonists not only reveals their neuroprotective potential but also provides new hope for PD treatment. By continuing to delve into these mechanisms, we hope to develop more effective therapies for PD, improving the quality of life for PD patients.
